# Cytomegalovirus (CMV) Pneumonitis: Cell Tropism, Inflammation, and Immunity

**DOI:** 10.3390/ijms20163865

**Published:** 2019-08-08

**Authors:** Luís Fonseca Brito, Wolfram Brune, Felix R. Stahl

**Affiliations:** 1 Institute of Clinical Chemistry and Laboratory Medicine, University Medical Center Hamburg-Eppendorf, Martinistraße 52, 20246 Hamburg, Germany; 2 Heinrich Pette Institute, Leibniz Institute for Experimental Virology, Martinistraße 52, 20251 Hamburg, Germany

**Keywords:** cytomegalovirus, CMV, HCMV, immunity, MCMV, NIF, lung, pneumonitis, pneumonia, tropism

## Abstract

Human cytomegalovirus (HCMV) is an opportunistic pathogen causing disease mainly in immunocompromised patients or after congenital infection. HCMV infection of the respiratory tract leads to pneumonitis in the immunocompromised host, which is often associated with a bad clinical course. The related mouse cytomegalovirus (MCMV) likewise exhibits a distinct tropism for the lung and thus provides an elegant model to study host-pathogen interaction. Accordingly, fundamental features of cytomegalovirus (CMV) pneumonitis have been discovered in mice that correlate with clinical data obtained from humans. Recent studies have provided insight into MCMV cell tropism and localized inflammation after infection of the respiratory tract. Accordingly, the nodular inflammatory focus (NIF) has been identified as the anatomical correlate of immune control in lungs. Several hematopoietic cells involved in antiviral immunity reside in NIFs and their key effector molecules have been deciphered. Here, we review what has been learned from the mouse model with focus on the microanatomy of infection sites and antiviral immunity in MCMV pneumonitis.

## 1. Introduction

Human Cytomegalovirus (HCMV, Human Herpesvirus 5) is a member of the β-herpesvirus subfamily and has a large double-stranded DNA genome of ~230 kilo base pairs [1]. Worldwide, HCMV infection is highly common, with seroprevalence rates ranging from 40 to nearly 100%. Primary infection is usually subclinical in healthy adults due to a complex antiviral immune response. However, the antiviral immune response cannot eliminate the virus, nor can it reliably prevent superinfection with additional HCMV strains or reactivation of the persisting virus. Thus, alterations in host immunity may allow for increased virus replication and manifestation of HCMV disease. Among the high-risk group are patients receiving immunosuppressive medication for prevention of organ transplant rejection, infection with immune-modulating pathogens such as human immunodeficiency virus (HIV), and infection in the early life period. In fact, congenital HCMV infection is the most frequent infectious cause of long-term neurological damage, such as sensorineural hearing loss and mental retardation [2]. Together, although HCMV is considered as an opportunistic pathogen, HCMV infection causes considerable clinical and economic burden [3]. Notably, HCMV exhibits a broad tissue tropism and thus various clinical symptoms have been described in patients suffering from Cytomegalovirus (CMV) disease. However, hepatitis, enterocolitis, retinitis, neurologic sequelae, and pneumonitis are among the most frequent organ manifestations [3].

The murine Cytomegalovirus (MCMV) has proven as an elegant tool to study principles of CMV infection in rodents that allow translation into the human system [4]. Several studies thus have been performed to study CMV pneumonitis in mice and defined the role of various immune cells to be involved in the anti-MCMV response. Moreover, modern imaging technology has led to identification of virus cell tropism in various organs. Finally, anatomical correlates of immune control have been defined in situ. These findings are in parallel to observations made in humans after HCMV infection and thus provide additional mechanistic insight into disease pathogenesis. Here, we focus on current knowledge about CMV infection of the respiratory tract and review what has been learned from studying the mouse cytomegalovirus (MCMV) in rodents.

## 2. Clinical Problem—HCMV Pneumonitis

### 2.1. High Risk Groups

Various clinical conditions have been associated with a high risk of HCMV infection leading to interstitial lung disease. Pneumonitis is the most common manifestation of HCMV infection in hematopoietic stem cell transplant (HSCT) recipients and a life threatening condition with high mortality rates [5,6]. Likewise, solid organ transplant recipients are at high risk to experience HCMV lung infection [7,8]. Despite antiviral prophylaxis HCMV pneumonitis may occur after lung transplantation and is associated with poor outcome [9]. HCMV lung infection is also a common disease of HIV infected patients [10] and HCMV pneumonitis can be the first manifestation of severe combined immunodeficiency (SCID) [11]. Moreover, neonatal HCMV pneumonitis often leads to chronic lung disease with fibrosis [12]. Interestingly, all of the aforementioned high-risk groups for HCMV pneumonitis show impairment in T cell immunity already indicating a relevant role for this immune cell type. Nevertheless, rare cases of HCMV pneumonitis have been observed in immune competent patients thus implying that also determinants of pathogenicity encoded by the virus may be causative for lung disease [13,14,15].

### 2.2. Clinical Symptoms and Diagnosis

HCMV lung infection can be asymptomatic under immunosuppression with clinical symptoms arising with recurring immune responses [16,17]. Symptoms are unspecific and include dry cough, breathlessness, dyspnoea on exertion, and fevers [18]. Radiological findings in HCMV pneumonitis are also rather unspecific and include diffuse interstitial infiltrates in chest radiography, and ground-glass opacity, small nodules and others in computed tomography [19]. Conclusively, the clinical and radiologic findings are typical for several causes of interstitial lung inflammation and this may cause difficulties for diagnosing HCMV pneumonitis [20]. Thus, the diagnosis of suspected HCMV pneumonitis is made by a combination of positive CMV serostatus together with detection of virus DNA in blood and/or bronchoalveolar lavage samples. However, the amount of HCMV DNA in bronchoalveolar fluids cannot differentiate between HCMV pneumonitis and asymptomatic pulmonary virus shedding [21]. Lung biopsy may allow to directly detect HCMV infection via immunohistopathology. Together, diagnosis of HCMV pneumonitis can be challenging and HCMV may also lead to asymptomatic infection of the lung.

### 2.3. Transmission and Ports of Entry

HCMV can be found in various body fluids [22,23] hence transmission pathways are likely manifold and include sexual as well as blood exposure. Systemic spread of the pathogen then may lead to secondary lung infection. In addition to transplacental infection in utero (congenital), newborns are at increased risk for perinatal infection via exposure to secretions in the birth canal or ingestion of virus-containing breast milk [24]. Interestingly, neonates and infants exhibit protracted shedding of virus in urine and saliva [25]. Accordingly, close contact to infected infants in day-care centers increases the risk of HCMV infection [26]. The latter observation implies that infection via the respiratory tract and HCMV pneumonitis may not only be the result of systemic virus dissemination within the host but also the result of direct airway exposure [27]. In fact, HCMV pneumonitis has been observed more frequently in perinatally than congenitally infected neonates supporting the notion that inhalation of the virus rather than a secondary manifestation after systemic spread causes infection of the respiratory tract [12]. Conclusively, besides dissemination to the lung after systemic infection, inhalation of contagious body fluids is a potentially relevant cause of HCMV infection and may lead to pneumonitis. Considering that HCMV infection does not necessarily lead to clinical symptoms together with the fact that pulmonary virus shedding can be observed in asymptomatic patients raises the question if infection via airway exposure is a currently underestimated transmission pathway of this pathogen.

### 2.4. HCMV Cell Tropism in the Respiraory Tract and Histopathological Findings

Cytomegalovirus mediates typical cytopathic effects with distinct morphological changes of infected host cells. Intra-nuclear inclusion bodies lead to owl’s eye appearance of these enlarged cells, thus allowing direct detection of CMV infection in histopathology [28]. Accordingly, HCMV-infection has been observed morphologically or via immunohistochemistry and in situ hybridization in alveolar epithelial cells (AEC), mesenchymal cells and macrophages (Mɸ) of the respiratory tract [29,30,31]. These HCMV-infected cells are accompanied by an inflammatory monocytic infiltrate [31]. Unfortunately, studies with comprehensive evaluation of histopathologic changes due to HCMV lung infection are rare. Accordingly, it is currently unknown how an acute HCMV infection manifests in the lung in the early phase and which immune cells are present at the site of infection to prevent overwhelming replication of the pathogen.

### 2.5. Summary

Pneumonitis may be a life-threatening manifestation of HCMV infection observed in patients with altered immunity and to a much lower extent in immunocompetent hosts. However, primary HCMV lung infections likely proceed with minimal symptoms in immunocompetent hosts and thus do not lead to medical consultation and diagnosis. The specific circumstances leading to symptomatic HCMV lung disease are not well defined as the alterations in immunity of the high-risk groups are diverse. Certainly, the high risk groups share alterations in T cell immunity and thus, T cell therapy in HCMV-infected HSCT patients is under clinical evaluation [32]. Hence, determinants of CMV lung infection have been studied in more detail in animal models which allow abrogation of specific immune functions and thus permit mechanistic insights into antiviral immunity in the respiratory tract. Several critical aspects of MCMV immune control after lung infection have been defined in the mouse model and these findings may be extrapolated to the human system.

## 3. Insights Obtained from the Animal Model—MCMV Pneumonitis

Infection of mice with MCMV is a well-established model to study antiviral mechanisms and infection of the respiratory tract shows fundamental similarities to HCMV infection. Already in 1978 Jordan described an interstitial pneumonitis caused by MCMV after intranasal application of the virus [33]. Moreover, MCMV established latent infection and reactivated in lung parenchyma after total-body gamma-irradiation following intraperitoneal application of the virus into neonates or subcutaneous (intraplantar) infection of adults [34,35,36]. The use of different genetically modified mice together with MCMV recombinants provide an elegant platform to dissect immune mechanisms involved in control of CMV lung infection. Accordingly, various studies provided insight into MCMV tropism to cells of the respiratory tract, leukocytes present at the site of infection, and relevant immune cells and effector molecules involved in control of lung infection.

### 3.1. MCMV Cell Tropism in the Respiratory Tract and Ports of Entry

**Figure 1:**
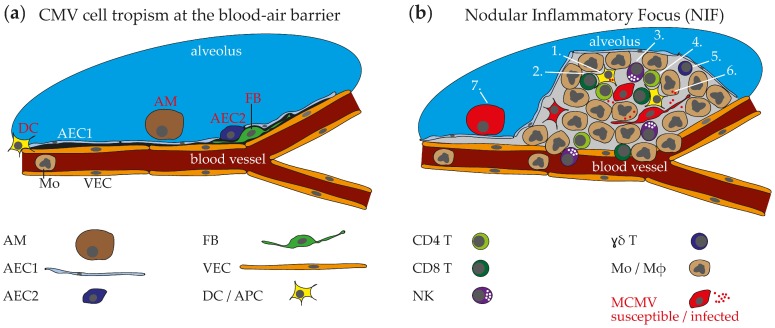
Microanatomy of mouse cytomegalovirus (MCMV) pneumonitis: (**a**) MCMV cell tropism at the blood-air barrier. Alveolar macrophages (AM), type 2 alveolar epithelial cells (AEC2), fibroblast-like (FB) stromal cells, and dendritic cells (DC) are susceptible to MCMV infection. Other blood-air barrier lining cells such as type 1 alveolar epithelial cells (AEC1) and lung vascular endothelial cells (VEC) were not found to be infected. (**b**) Schematic illustration of a Nodular Inflammatory Focus (NIF) as the site of local anti-MCMV immune response in the lung. NIFs comprise infected and immune cells involved in control of infection. **1**. Antigen-presenting cells (APC) such as DCs present viral antigens to T cells, **2**. CD8 T cells recognize peptide on Major histocompatibility complex (MHC)-I cross-presented by DCs and secrete Interferon-gamma (IFN-ɣ), **3**. Natural killer (NK) cells probably directly recognize infected cells and mediate immunity via perforin and IFN-ɣ, **4**. CD4 T cells recognize peptide on MHC-II presented by DCs and macrophages (Mɸ) and secrete IFN-ɣ and Tumor necrosis factor-α (TNF-α), **5**. ɣδ T cells are supposed to be present in NIFs and may produce cytokines such as IFN-ɣ, **6**. Monocytes (Mo) and Mɸs phagocytose infectious particles and may present antigen to CD4 T cells, **7**. How AMs may be involved in MCMV control is currently not known.

The lung parenchyma mainly consists of blood vascular endothelial cells (VEC) and AECs. Together, these cells build up a very thin blood-air barrier that allows gas exchange in the respiratory tract. Moreover, fibroblast (FB)-like stromal cells and immune cells have been described to be localized in the lung interstitium [37]. The alveoli lining epithelium is constantly renewed to allow permanent tissue integrity. In this respect, type 2 AECs (AEC2) are precursors of type 1 AECs (AEC1) and the latter outnumbers the first in absolute cell numbers and cell membrane surface area [38]. Finally, alveolar macrophages (AM) patrol the alveoli to take up airborne particles and thereby allow undisturbed gas exchange [39]. Interestingly, systemic or peripheral application (intravenous, intraperitoneal, or subcutaneous) as well as inoculation of the virus directly into the respiratory tract (intranasal, intratracheal, or laryngopharyngeal) leads to infection of the lung. This indicates that the blood-air barrier lining cells of the lung are both susceptible to MCMV infection, from the air as well as from the blood side (Figure 1a). Indeed, in accordance with findings obtained from human tissue, pro-surfactant protein-C^+^ AEC2s and CD11c^+^Siglec-F^+^ AMs have been identified to be primary targets of MCMV after inoculation of the virus from the respiratory side and have previously been suggested as targets after systemic infection of animals with immunodeficiency [40,41,42,43,44,45]. However, infection of AMs depends on the expression of an intact MCMV-encoded chemokine 2 (MCK2) as an MCMV strain with a truncated version of the protein (MCK2^−^) did not infect this cell type [43]. Interestingly, MCK2 is part of the gH/gO/MCK2 entry complex and also has been shown to promote infection of liver macrophages [46]. Finally, subsequent infection of cells with FB morphology has been described in vivo [42] and this was confirmed by the observation that gp38^+^PDGFRA^+^PDGFRB^+^CD31^−^ stromal cells isolated from lungs are highly susceptible to MCMV infection in vitro [47]. Considering the various cell types that are susceptible to MCMV infection, the virus has elaborate options to propagate within the lung via cell-to-cell spread. However, as MCMV infection interferes with expression of cell membrane proteins, caution is required when new virus cell targets are identified by antibody staining of cell surface markers [48,49,50,51]. Due to the close contact to blood circulation, the virus likely uses multiple pathways to disseminate from the lung to other organs and vice versa. A recent study reported dendritic cells (DC) to be involved in systemic spread from the lung exploiting the lymphatic system [52]. However, MCMV is perfectly capable of spreading to the salivary glands after intranasal infection in total-body gamma-irradiated mice when most of the leukocytes are absent [47]. Similarly, *Rag2*^−/−^*Il2rg*^−/−^ which lack lymph nodes [53] show unrestricted dissemination of a MCK2^−^ virus to salivary glands, liver, spleen, kidneys, and other organs after intranasal infection (own unpublished observation). MCMV primarily targets the lung parenchyma and the nasal (olfactory) epithelium after intranasal infection [54]. Accordingly, the virus may spread from either the lungs or the olfactory epithelium to other organs independent of DCs and lymph nodes after intranasal application. Less is known on infection of the respiratory tract after systemic or peripheral application of the virus. However, infection of transgenic mice with an endothelial-specific receptor tyrosine kinase (Tie2) promoter driven expression of Cre recombinase with an MCMV-flox recombinant virus leads to infection of the lung indicating that VECs are a direct target after systemic infection [55]. However, Tie2 expression is not VEC-specific but also found in pericytes which share similarities with FBs [56]. In this respect, MCMV tropism for lung blood VECs needs further formal verification via histology. Studies of other possible entry pathways, e.g., infection by immigrating virus-containing monocytes from the blood circulation, are currently not available. In summary, lungs are a robust target of MCMV infection independent from host immunity or infection route and thus frequently involved when a host encounters this pathogen.

### 3.2. Nodular Inflammatory Focus as Site of Immune Control in Lungs

MCMV infection of lung parenchyma cells is followed by a local cell-to-cell spread of the virus within the organ tissue and thus leads to focal clusters of infected cells in juxtaposition with each other. The appearance of localized multiple infected cells is comparable to observations made in vitro where MCMV leads to formation of plaques due to its cytopathic effect in susceptible cells [42,47]. However, formation of plaque-like structures in vivo attracts leukocytes into interstitial regions of the lung. Thus, three weeks after virus administration CD3ε^+^ T cells have been detected next to MCMV-infected cells in inflammatory foci of the lung after bone marrow transplantation into total-body gamma-irradiated mice [41,57]. Similarly, a cluster of MCMV infected cells were described in lungs of neonatal mice after intraperitoneal infection [58]. A more comprehensive study in neonatal mice provided insights into the diversity of immune cells present at the site of infection [42]. In this study, the authors found many myeloid cells to be present at the site of infection—a fact which has not been reported in previous studies. Due to the abundance of myeloid cells and resulting granuloma-like appearance of these structures the term “nodular inflammatory focus” (NIF) was adopted from the description of histopathological findings in immunocompromised patients with HCMV pneumonitis [42,59]. In a following study by the same group, NIFs were more precisely described in adult lungs after intranasal MCMV infection [47]. As MCMV initially infects many cells at different sites within the organ, multiple NIFs are distributed throughout the lung parenchyma. Immune cells found in NIFs are of lymphoid as well as myeloid origin (Figure 1b). CD3^+^CD4^+^ and CD3^+^CD8^+^ T, natural killer (NK), and B cells are present in NIFs of neonatal and adult lungs [42,47]. Moreover, ɣδ T cells have been described to enrich after MCMV lung challenge and it is likely that they also accumulate in NIFs. However, their location within the organ during an MCMV challenge has not been studied yet [60]. The myeloid cells present in NIFs have been positively stained via immunohistochemistry for CD11b, F4/80, CD11c, CD169 and CD103 indicating presence of a mixed population of cells including monocytes (Mo), and potential antigen-presenting cells (APC) such as macrophages (Mɸ) and DCs. The presence of NIFs is a temporally dynamic phenomenon as the immune cells found in NIFs are involved in control of MCMV infection [41,42,47,57]. Thus, these structures disappear in immunocompetent hosts after the virus is controlled [42,47], although peribronchial antigen-specific T cells can still be found in latently infected lungs [61]. In contrast, presence of NIFs is extended in lungs of infected immunocompromised hosts such as T cell-deficient and neonatal mice [42,47]. In this situation, infection may lead to morbidity and/or prolonged virus replication. Similarly, MCK2^+^ MCMV infection leads to more inflammation and increased NIF size if compared to MCK2^−^ virus and this was accompanied with higher virus loads [43]. Accordingly, understanding the antiviral mechanisms operating within NIFs is crucial to predict consequences for lung parenchyma integrity and immunopathology.

### 3.3. Immune Cells in MCMV Pneumonitis

Various models have been used to study antiviral immunity against MCMV infection in mice including different infection routes, virus strains, mouse strains, and time-points of analysis post infection. The ample amount of data provides strong evidence for the role of various immune cells involved in control of MCMV infection. However, growing evidence for tissue-specific differences in immune cells and their effector mechanisms requires assessment of antiviral immunity in an organ-specific context with focus on mechanisms relevant locally at the site of infected tissue [62]. Unfortunately, lung virus loads have been measured mainly after systemic MCMV infection. Thus, the effects observed in the lung could be the result of indirect non-lung antiviral immunity and a secondary process of decreased dissemination from other organs. Here, we summarize what is known on anti-MCMV immune responses in the respiratory tract hypothesizing that one can correlate effects on lung virus titers to modes of action in situ within NIFs. Table 1 surveys hematopoietic cells implicated in antiviral immunity with ascribed function in MCMV pneumonitis independent of the route of infection. 

#### 3.3.1. αβ T Cells

The role of immune cells in control of MCMV lung infection was first demonstrated by application of antiserum to murine lymphocytes in MCMV-infected mice. Apparently, this antiserum lead to in vivo depletion of lymphocytes and caused fatal MCMV disease with high viral loads in the lungs of MCMV-infected animals [45]. Similarly, administration of the immunosuppressive chemotherapeutic agent cyclophosphamide or total-body gamma-irradiation increased virus loads in lungs of mice infected with MCMV [40,77]. Both procedures interfere non-specifically with haematopoiesis. Thus, although these early experiments established that immune cells are critical for control of MCMV infection, the role of different immune cell subsets could not be extrapolated from that data. More mechanistic insight was provided by adoptive transfer of antigen-experienced CD8 T cells into total-body gamma-irradiated mice. This procedure reduced virus loads in lungs and established the predominant role of CD8 T cells in control of MCMV lung infection [40,63]. Moreover, antibody-mediated CD4 T cell depletion in vivo increased virus titers in the lungs [66]. However, the effects of CD4 T cells seemed to be of minor importance in comparison to those of CD8 T cells. From these early studies various models were used to more precisely dissect the role of CD4 and CD8 T cells during MCMV challenge. 

In a model of syngeneic bone marrow transplantation T cells were found in proximity to MCMV-infected lung parenchymal cells in histology sections [57,65]. Apparently, this T cell population contained MCMV-specific CD8 T cells with ex vivo cytotoxic capacity. Moreover, T cell reconstitution and presence of CD8 T cells in lungs temporally coincided with reduction of virus loads implicating direct antiviral effects of MCMV-specific CD8 T cells in the lung parenchyma. In the same model, in vivo depletion of CD8 T cells had a stronger effect on lung virus loads than depletion of CD4 T cells only [41,64]. However, long-term in vivo depletion of CD8 T cells still lead to effective control of MCMV in lungs when CD4 T cells were present [67]. More recent studies found MCMV-specific CD8 T cells to expand after both intraperitoneal and intranasal infection [78,79]. MCMV infection of athymic nude mice which lack T cells lead to progressive virus replication with pneumonitis [80]. Thus, CD4 and CD8 T cells may substitute for each other during chronic infection but simultaneous absence of both leads to severe disease.

Antigen-specific CD4 T cells were detected in lungs of MCMV-infected C57BL/6 mice in the acute but not latent infection phase [68]. The same group generated transgenic mice with a CD4 T cell receptor specific for a sequence of the MCMV encoded M25 protein. Adoptive transfer of antigen-experienced CD4 T cells obtained from these mice were protective in the lung of total-body gamma-irradiated mice [70]. Moreover, this study highlighted the role of Interferon gamma (IFN-ɣ) secreted by CD4 T cells whereas perforin-mediated cytotoxicity was not relevant for the control of lung infection. Most MCMV-infected cells in the lung do not express MHC-II and thus CD4 T cells cannot directly recognize these cells for directional degranulation of enzymes. Interestingly, adoptive transfer of low numbers of M25-specific CD4 together with M38-specific CD8 T cells was more effective than each cell type on its own in reducing virus titers in lungs. Thus, this study elegantly confirmed the previous assumptions that CD4 and CD8 T cells control MCMV lung infection cooperatively. Accordingly, the increased virus loads in NIFs of lungs after intranasal infection of T and B cell deficient *Rag2*^−*/*−^ mice could be rescued by adoptive transfer of polyclonal antigen inexperienced CD4 together with CD8 T cells [47]. In this model CD8 T cells partially reduced virus loads in lungs whereas transfer of purified CD4 T cells was not protective. Importantly, adoptive transfer of CD4 and CD8 T cells obtained from perforin-deficient *Prf1*^−*/*−^ into *Rag2*^−*/*−^ mice led to the same virus loads as if compared to transfer of cells obtained from wildtype mice. In contrast, higher virus loads in lungs were found when cells were obtained from *Ifng*^−*/*−^ mice and transferred into *Rag2*^−*/*−^ mice. Thus, not only CD4 T cells but also CD8 T cells acted independent from perforin-mediated cytotoxicity, but both applied IFN-ɣ. Cytomegaloviruses are notorious for interfering with immune responses. In particular, MCMV encoded m06 and m152 prevent antigen presentation on MHC-I molecules of infected cells [48,81]. Thus, CD8 T cells may not recognize their cognate antigen presented on MCMV-infected cells and therefore lack directionality to secrete perforin and granzyme granules into an immunological synapse [82]. Accordingly, paracrine impact of effector molecules secreted by T cells, such as IFN-ɣ, seem to be of higher relevance than direct cytotoxicity to control MCMV infection in the respiratory tract. Moreover, CD4 T cell mediated control of MCMV lung infection is increased in absence of IL-10, likely via increased production of IFN-ɣ and TNF-α [69]. In summary, there is ample evidence for T cells to be critical in control of MCMV lung infection. Although CD8 T cells seem to be more dominantly involved in control of acute infection, there is an important role for both CD4 and CD8 T cells.

#### 3.3.2. NK Cells

In 1984 Bukowski et al. proposed natural killer (NK) cells to be involved in reducing virus loads in lungs of MCMV-infected C57BL/6 mice. In vivo depletion of NK cells with anti-asialo ganglioside (GM1) antibodies increased MCMV pathogenesis and virus titers [71]. However, it needs to be mentioned that GM1 is expressed by various immune cells thus lacking specificity for NK cells. Nevertheless, the role of NK cells for control of MCMV lung infection was confirmed later utilizing anti-NK1.1 antibodies for cell depletion [72]. Both, IFN-ɣ as well as perforin were proposed to be involved in lung protection. Accordingly, *Rag2*^−*/*−^*Ifng*^−*/*−^ and *Rag2*^−*/*−^*Prf1*^−*/*−^ mice both had more infected cells in lungs than *Rag2*^−*/*−^ mice and IFN-ɣ producing NK cells could be isolated from MCMV-Δm157 infected lungs suggesting a role for these cells when T cells are absent [47]. In the same study, application of anti-NK1.1 antibodies into wildtype mice had only a minor effect on virus titers after intranasal MCMV-Δm157 infection. Thus, host immune status, infection route, and observation time point after infection most likely affect the antiviral role of NK cells in lungs. Interpretation of studies on natural killer (NK) cell responses against MCMV in lung infection requires caution as expression of the activating NK cell receptor Ly49H varies between mouse strains. In this respect, NK cells of the commonly used mouse strain C57BL/6 express Ly49H and thus C57BL/6 mice were considered to be more resistant to MCMV infection than “susceptible” Ly49H^−^ BALB/c mice. Moreover, the interaction of Ly49H^+^ NK cells with MCMV-infected cells can be subverted by targeted deletion of the MCMV encoded Ly49H ligand m157 [83]. Accordingly, in studies with MCMV-m157^+^ of C57BL/6 mice usually stronger NK cell-mediated antiviral effects were observed than with MCMV-Δm157 mutants. In another study, NK cells have been proposed to enhance CD4 T cell responses in an Il-10 deficient host [69]. Conclusively, there is ample data supporting a role for these cells in control of MCMV pneumonitis but future studies should aim to more specifically investigate how NK cells mediate their antiviral effects in the lung.

#### 3.3.3. Myeloid Cells

Myeloid cells are highly abundant in NIFs and causative for their granuloma-like appearance. Interestingly, NIFs arise independently from the presence of lymphocytes and can be robustly detected e.g., in *Rag2*^−*/*−^*Il2rg*^−*/*−^ lymphopenic mice [47]. Mouse models with a specific lack of myeloid cells are currently not available. Thus, studying the role of this diverse cell population is difficult. Nevertheless, comparison of total-body gamma-irradiated mice, where hardly any hematopoietic cells can be detected, to *Rag2*^−*/*−^*Il2rg*^−*/*−^ lymphopenic mice may allow investigation of myeloid cells. In fact, viral titers are increased in lungs of total-body gamma-irradiated mice if compared to *Rag2*^−*/*−^*Il2rg*^−*/*−^ mice [47]. This in vivo data indicates a role for myeloid cells in interfering with MCMV infection. However, gamma-irradiation may have other effects on non-hematopoietic parenchymal cells that could be causative for increased virus titres in lungs in vivo. Nevertheless, in the presence of IFN-ɣ, combined culture of primary lung stromal cells together with lung Mɸ interfered with MCMV plaque formation in vitro. A direct antiviral effect of IFN-ɣ was also observed later after infection when myeloid cells were absent [47]. Thus, IFN-ɣ stimulated myeloid cells possibly contain MCMV lung infection in situ as described in vitro. Remnants of cells infected with MCMV have been observed in myeloid cells within neonatal NIFs indicating that these cells phagocytose infected cells and consequently eliminate virions from the local infection site [42]. Some cells present in NIFs have been identified as antigen presenting cells, such as CD169^+^ or CD103^+^ DCs, and they may be involved in the unanticipated feature of NIFs as ectopic T cell priming sites. Moreover, these cells are likely important for cross-presentation of peptide to CD8 T cells [84] in the effector phase thus facilitating paracrine cytokine secretion of effector molecules by T cells, such as IFN-ɣ, to interfere with MCMV replication. A role for neutrophils in MCMV lung infection has been demonstrated by in vivo depletion of Ly6G^+^ cells [74]. Virus titers were decreased in lungs four days after systemic infection and further functional analysis revealed TNF-related apoptosis-inducing ligand (TRAIL) to be the responsible molecule involved in neutrophil antiviral activity. A more recent study suggested CD11b^+^ DCs to be involved in allergic airway disease [85]. In this study, intratracheal MCMV infection was combined with application of ovalbumin. Subsequent aerosol challenge with the same protein led to increased inflammation in the MCMV-infected group. Nevertheless, myeloid cells are also direct targets of MCMV and infection of AMs led to higher virus titres in lungs [43]. Interestingly, in vivo depletion of AMs via intranasal administration of clodronate liposomes reduced MCMV titers possibly by reduction of cells that could be infected primarily. However, the mechanism behind this finding is not known and it is also not known if AMs themselves contribute to immunity in MCMV pneumonitis. Finally, myeloid cells have been proposed to be important for virus dissemination [52]. Thus, the various subsets of myeloid cells may have versatile and opposed effects on MCMV infection and clearly need more attention in future studies.

#### 3.3.4. Antibodies, ɣδ T Cells, and More

Absence of B cells in *Ighm*^−*/*−^ mice had no impact on control of MCMV in lung NIFs during the acute infection phase [47]. However, adoptive transfer of antigen-exposed B cells into *Rag1*^−*/*−^ mice lead to measurable antibody titres and was protective later after infection [75]. Similarly, ɣδ T cells isolated from infected mice were protective in the lungs of MCMV-infected *Rag1*^−*/*−^ mice [73]. Moreover, lungs of *Tcrd*^−*/*−^ mice had higher viral loads than wildtype mice at three days post infection indicating an innate antiviral effect against MCMV. Accordingly, progressively increasing numbers of ɣδ T cells could be detected in lungs of *Tcra*^−*/*−^ mice after systemic infection with MCMV supporting the hypothesis of infection-induced expansion of these cells [60]. In a study by Ebert et al. peritoneal cavity mast cells have been proposed as direct targets of MCMV infection and their degranulation affected CD8 T cell responses in the respiratory tract [76]. However, it is not known if lung-resident mast cells also affect MCMV lung infection in situ. Other cells which have been described to have antiviral effects such as plasmacytoid dendritic cells (pDC) or non-NK cell innate lymphoid cells (ILC) currently lack evidence to play a role in MCMV pneumonitis.

#### 3.3.5. Integral Model of Anti-MCMV Immune Response in NIFs

In summary, several relevant antiviral immune mechanisms in control of MCMV lung infection have been discovered. APCs in NIFs present MCMV peptides to CD4 and CD8 T cells to facilitate anti-MCMV T cell immunity. This mechanism seems to be of particular importance for CD8 T cells as immune evasion features of the virus interfere with direct recognition of the infected cells. Hence, cross-presentation of peptide by APCs is required to maintain the CD8 T cell effector response. Accordingly, the main antiviral effects mediated by T cells in NIFs likely rely on cytokine secretion. In this respect, IFN-ɣ has been shown to be of relevance to directly inhibit virus spread and stimulate antiviral effects of Mɸ’s. IFN-ɣ is also produced by NIF NK cells during the early infection phase but these cells also likely directly recognize and kill infected cells in a perforin dependent manner. Under specific circumstances ɣδ T cells, neutrophils and antibodies may substitute or add to these major antiviral effects. Conclusively, the complex network of various immune cells present in NIFs synergistically interferes with overwhelming MCMV infection and contain the virus in focal sites of inflammation in the immune competent host. Consequently, MCMV infection may lead to symptomatic lung disease if one or more units of these antiviral mechanisms are altered.

### 3.4. Summary

In conclusion, virus replication in lungs and pneumonitis are frequently observed after infection of mice with MCMV. The virus may spread to the lung during systemic infection or spread from the lung to other organs after primary lung infection. AECs, AMs, and FBs have been identified as major targets of the virus after intranasal administration. CD4 and CD8 T cells are found in NIFs, crucial to control MCMV pneumonitis, and mediate their antiviral effects via cytokines. Other lymphocytes such as NK cells as well as myeloid cells are also present in NIFs and contribute to containment of the infection. 

## 4. Perspective

Despite the ample data available on anti-MCMV immune responses in vivo there are several critical questions unanswered that need to be addressed in future studies. Age-related differences in MCMV immunity need to be defined to explain the prolonged lung infection in neonates. The role of transmission of the virus via airway exposure needs further attention to sensitize physicians for this route of infection. Genome analysis of HCMV strains need to be acquired to decipher determinants of pathogenicity.

### 4.1. Age-Related Differences in CMV Pneumonitis

CMV is the most relevant infectious cause of permanent disabilities in human children and HCMV has been shown to persist exceedingly long after infection if compared to adults [86]. Accordingly, lung infection of neonatal mice with MCMV had a prolonged course of infection if compared to lung infection of adults [42]. Moreover, systemic (intraperitoneal) MCMV infection of neonatal mice led to high recurrence from latency whereas recurrence was lower in adult mice after peripheral (intraplantar) infection [35]. The reasons for this are currently not known. Interestingly, adoptive transfer of SIINFEKL-specific CD8 T cells to neonatal mice infected with a SIINFEKL-expressing MCMV was not protective in lung NIFs [42]. Although the transferred cells could be detected in high numbers in NIFs and proliferated in an antigen-dependent manner, they could not decrease virus titres in the infected lung tissue. Thus, this data contradicts findings from infected adult mice using other models of lymphopenia and adoptive transfer of CD8 T cells [40,47,63,65]. The cellular and molecular mechanisms behind this need further analysis to understand why the early life is such an advantageous window for CMV replication and consequential pathogen shedding to other hosts. In particular, the quality and phenotype of anti-CMV T cell responses in lungs during the early life need to be assessed and quantified. Moreover, age-related differences in the function of other immune cells such as NK, ɣδ T and other innate lymphoid cells as well as the role of maternal and infant antibodies need to be studied to define their contribution in control of neonatal CMV lung infection. Sophisticated MCMV infection models are needed to study principles of the underlying mechanism to subsequently confirm robust findings in any of the precious human samples collected from young children and neonates.

### 4.2. CMV Transmission via Airway Exposure

It needs to be addressed if primary infection of the respiratory tract via inhalation of contagious saliva is an important transmission pathway as suggested [27]. In this context, infection of the nasal (olfactory) epithelium and secondary systemic spread has been suggested as an additional infection route after intranasal MCMV application [54]. Thus, the virus may use multiple ways to invade the host after airway exposure. It is currently unclear if lung infection in the immune competent host leads to symptoms and thus can be robustly detected in epidemiologic studies. Interestingly, direct application of MCMV into the neonatal lung does not lead to the high morbidity as observed after intraperitoneal infection [42,87]. Thus, infection via the respiratory tract may be a port of entry used by this opportunistic pathogen to spread from host to host especially during the early life period. Sensitive and specific correlates of lung CMV infection need to be established to detect and differentiate tissue-specific virus disease. Accordingly, new biomarkers need to be generated to address this question in a practical and ethical approach. Again, infection of mice with MCMV is likely the most attractive and feasible model to fill these gaps of knowledge with data. 

### 4.3. CMV Determinants of Pathogenicity

It is currently not known if specific CMV genotypes are relevant for lung infection and which genetic determinants would be advantageous for the virus to spread between hosts. Thus, clinical CMV isolates obtained from lung infected hosts should be analysed for such genetic determinants. Definition of genetic determinants of pathogenicity would allow predicting CMV lung disease in the clinical setting. Inter-species homologous genetic regions may be studied in mice to understand any mechanism behind. 

## 5. Conclusions

After more than four decades of research, many parallels between HCMV and MCMV pneumonitis have been identified. Both viruses (i) show tropism to similar host cells such as AECs, AMs and other FB-like cells, (ii) lead to comparable histopathology with focal influx of myeloid cells to the site of infection, (iii) and are controlled by the immune-competent host but lead to disease when T cell antiviral immunity is altered. These central common features between the two different species and corresponding viruses further support the use of the MCMV model to understand anti-CMV immune responses in humans. Experimental studies in mice provided insight into which immune cells act directly at the site of infection to contain the pathogen in NIFs. Key effector molecules involved in control of virus infection have been identified, providing the basis for improvement of therapeutic approaches. Moreover, the combination of the findings obtained from the mouse model together with epidemiological data raise the question if HCMV pneumonitis is an underdiagnosed primary manifestation of this virus infection. MCMV pneumonitis is mostly asymptomatic in resistant mice even in the early life period and human infants are known to shed high virus loads via saliva. Thus, lung infection with mild asymptomatic pneumonitis after inhalation of contagious saliva may be an underestimated infection route of HCMV that needs to be evaluated in future studies.

## Figures and Tables

**Table 1:** Immune cells and their antiviral function in MCMV pneumonitis.

Cell Type	Cell Present in NIF	Function	References
CD8 T cell	yes	IFN-ɣ	[40,41,57,63,64,65]
CD4 T cell	yes	IFN-ɣ, TNF-α	[41,47,64,66,67,68,69,70]
NK cell	yes	Perforin, IFN-ɣ	[47,69,71,72]
ɣδ T cell	?	IFN-ɣ?	[60,73]
Mɸ	yes	Phagocytosis	[42,47]
DC	yes	Antigen presentation	[42]
Neutrophil	?	TRAIL	[74]
B cell / plasma cell	yes / ?	Antibodies	[75]
Mast cell	?	Indirect via CD8 T cell	[76]

## References

[1] Arvin A., Campadelli-Fiume G., Mocarski E., Moore P.S., Roizman B., Whitley R., Yamanishi K. (2007). Human Herpesviruses: Biology, Therapy, and Immunoprophylaxis.

[2] Rawlinson W.D., Boppana S.B., Fowler K.B., Kimberlin D.W., Lazzarotto T., Alain S., Daly K., Doutre S., Gibson L., Giles M.L. (2017). Congenital cytomegalovirus infection in pregnancy and the neonate: Consensus recommendations for prevention, diagnosis, and therapy. Lancet Infect. Dis..

[3] Griffiths P.D. (2012). Burden of disease associated with human cytomegalovirus and prospects for elimination by universal immunisation. Lancet Infect. Dis..

[4] Reddehase M.J., Lemmermann N.A.W. (2018). Mouse Model of Cytomegalovirus Disease and Immunotherapy in the Immunocompromised Host: Predictions for Medical Translation that Survived the “Test of Time”. Viruses.

[5] Schmidt G.M., Horak D.A., Niland J.C., Duncan S.R., Forman S.J., Zaia J.A. (1991). A randomized, controlled trial of prophylactic ganciclovir for cytomegalovirus pulmonary infection in recipients of allogeneic bone marrow transplants; The City of Hope-Stanford-Syntex CMV Study Group. N. Engl. J. Med..

[6] Boeckh M., Ljungman P. (2009). How we treat cytomegalovirus in hematopoietic cell transplant recipients. Blood.

[7] Fishman J.A. (2007). Infection in solid-organ transplant recipients. N. Engl. J. Med..

[8] Hill R.B., Rowlands D.T., Rifkind D. (1964). Infectious Pulmonary Disease in Patients Receiving Immunosuppressive Therapy for Organ Transplantation. N. Engl. J. Med..

[9] Snyder L.D., Finlen-Copeland C.A., Turbyfill W.J., Howell D., Willner D.A., Palmer S.M. (2010). Cytomegalovirus pneumonitis is a risk for bronchiolitis obliterans syndrome in lung transplantation. Am. J. Respir. Crit. Care Med..

[10] Wallace J.M., Hannah J. (1987). Cytomegalovirus pneumonitis in patients with AIDS. Findings in an autopsy series. Chest.

[11] Szczawinska-Poplonyk A., Jonczyk-Potoczna K., Ossowska L., Breborowicz A., Bartkowska-Sniatkowska A., Wachowiak J. (2014). Cytomegalovirus pneumonia as the first manifestation of severe combined immunodeficiency. Cent. Eur. J. Immunol..

[12] Coclite E., di Natale C., Nigro G. (2013). Congenital and perinatal cytomegalovirus lung infection. J. Matern Fetal. Neonatal Med..

[13] Cunha B.A., Pherez F., Walls N. (2009). Severe cytomegalovirus (CMV) community-acquired pneumonia (CAP) in a nonimmunocompromised host. Heart Lung.

[14] Grilli E., Galati V., Bordi L., Taglietti F., Petrosillo N. (2012). Cytomegalovirus pneumonia in immunocompetent host: Case report and literature review. J. Clin. Virol..

[15] Goncalves C., Cipriano A., Videira Santos F., Abreu M., Mendez J., Sarmento E.C.R. (2018). Cytomegalovirus acute infection with pulmonary involvement in an immunocompetent patient. IDCases.

[16] Grundy J.E., Shanley J.D., Griffiths P.D. (1987). Is Cytomegalovirus Interstitial Pneumonitis in Transplant Recipients an Immunopathological Condition. Lancet.

[17] Barry S.M., Johnson M.A., Janossy G. (2000). Cytopathology or immunopathology? The puzzle of cytomegalovirus pneumonitis revisited. Bone Marrow Transplant.

[18] Rafailidis P.I., Mourtzoukou E.G., Varbobitis I.C., Falagas M.E. (2008). Severe cytomegalovirus infection in apparently immunocompetent patients: A systematic review. Virol. J..

[19] Moon J.H., Kim E.A., Lee K.S., Kim T.S., Jung K.J., Song J.H. (2000). Cytomegalovirus pneumonia: High-resolution CT findings in ten non-AIDS immunocompromised patients. Korean J. Radiol..

[20] Restrepo-Gualteros S.M., Gutierrez M.J., Villamil-Osorio M., Arroyo M.A., Nino G. (2019). Challenges and Clinical Implications of the Diagnosis of Cytomegalovirus Lung Infection in Children. Curr. Infect. Dis. Rep..

[21] Pinana J.L., Gimenez E., Gomez M.D., Perez A., Gonzalez E.M., Vinuesa V., Hernandez-Boluda J.C., Montoro J., Salavert M., Tormo M. (2019). Pulmonary cytomegalovirus (CMV) DNA shedding in allogeneic hematopoietic stem cell transplant recipients: Implications for the diagnosis of CMV pneumonia. J. Infect..

[22] Lippold S., Braun B., Kruger F., Harms M., Muller J.A., Gross R., Munch J., von Einem J. (2019). Natural Inhibitor of Human Cytomegalovirus in Human Seminal Plasma. J. Virol..

[23] Ziemann M., Thiele T. (2017). Transfusion-transmitted CMV infection—Current knowledge and future perspectives. Transfus. Med..

[24] Azenkot T., Zaniello B., Green M.L., Selke S., Huang M.L., Magaret A., Wald A., Johnston C. (2018). Cytomegalovirus shedding from breastmilk and mucosal sites in healthy postpartum women: A pilot study. J. Med. Virol..

[25] Cannon M.J., Stowell J.D., Clark R., Dollard P.R., Johnson D., Mask K., Stover C., Wu K., Amin M., Hendley W. (2014). Repeated measures study of weekly and daily cytomegalovirus shedding patterns in saliva and urine of healthy cytomegalovirus-seropositive children. BMC Infect. Dis..

[26] Adler S.P. (1988). Molecular epidemiology of cytomegalovirus: Viral transmission among children attending a day care center, their parents, and caretakers. J. Pediatr..

[27] Jackson J.W., Sparer T. (2018). There Is Always Another Way! Cytomegalovirus′ Multifaceted Dissemination Schemes. Viruses.

[28] Herriot R., Gray E.S. (1994). Images in clinical medicine. Owl’s-eye cells. N. Engl. J. Med..

[29] Sinzger C., Grefte A., Plachter B., Gouw A.S., The T.H., Jahn G. (1995). Fibroblasts, epithelial cells, endothelial cells and smooth muscle cells are major targets of human cytomegalovirus infection in lung and gastrointestinal tissues. J. Gen. Virol..

[30] Andrade Z.R., Garippo A.L., Saldiva P.H., Capelozzi V.L. (2004). Immunohistochemical and in situ detection of cytomegalovirus in lung autopsies of children immunocompromised by secondary interstitial pneumonia. Pathol. Res. Pract..

[31] Restrepo-Gualteros S.M., Jaramillo-Barberi L.E., Gonzalez-Santos M., Rodriguez-Martinez C.E., Perez G.F., Gutierrez M.J., Nino G. (2014). Characterization of cytomegalovirus lung infection in non-HIV infected children. Viruses.

[32] Mui T.S., Kapp M., Einsele H., Grigoleit G.U. (2010). T-cell therapy for cytomegalovirus infection. Curr. Opin. Organ Transpl..

[33] Jordan M.C. (1978). Interstitial pneumonia and subclinical infection after intranasal inoculation of murine cytomegalovirus. Infect. Immun..

[34] Balthesen M., Messerle M., Reddehase M.J. (1993). Lungs are a major organ site of cytomegalovirus latency and recurrence. J. Virol..

[35] Reddehase M.J., Balthesen M., Rapp M., Jonjic S., Pavic I., Koszinowski U.H. (1994). The conditions of primary infection define the load of latent viral genome in organs and the risk of recurrent cytomegalovirus disease. J. Exp. Med..

[36] Steffens H.P., Kurz S., Holtappels R., Reddehase M.J. (1998). Preemptive CD8 T-cell immunotherapy of acute cytomegalovirus infection prevents lethal disease, limits the burden of latent viral genomes, and reduces the risk of virus recurrence. J. Virol..

[37] Mattoli S. (2015). Involvement of fibrocytes in asthma and clinical implications. Clin. Exp. Allergy.

[38] Stone K.C., Mercer R.R., Gehr P., Stockstill B., Crapo J.D. (1992). Allometric relationships of cell numbers and size in the mammalian lung. Am. J. Respir.Cell Mol. Biol..

[39] Bohmwald K., Espinoza J.A., Pulgar R.A., Jara E.L., Kalergis A.M. (2017). Functional Impairment of Mononuclear Phagocyte System by the Human Respiratory Syncytial Virus. Front. Immunol..

[40] Reddehase M.J., Weiland F., Munch K., Jonjic S., Luske A., Koszinowski U.H. (1985). Interstitial murine cytomegalovirus pneumonia after irradiation: Characterization of cells that limit viral replication during established infection of the lungs. J. Virol..

[41] Podlech J., Holtappels R., Pahl-Seibert M.F., Steffens H.P., Reddehase M.J. (2000). Murine model of interstitial cytomegalovirus pneumonia in syngeneic bone marrow transplantation: Persistence of protective pulmonary CD8-T-cell infiltrates after clearance of acute infection. J. Virol..

[42] Stahl F.R., Heller K., Halle S., Keyser K.A., Busche A., Marquardt A., Wagner K., Boelter J., Bischoff Y., Kremmer E. (2013). Nodular inflammatory foci are sites of T cell priming and control of murine cytomegalovirus infection in the neonatal lung. PLoS Pathog..

[43] Stahl F.R., Keyser K.A., Heller K., Bischoff Y., Halle S., Wagner K., Messerle M., Forster R. (2015). Mck2-dependent infection of alveolar macrophages promotes replication of MCMV in nodular inflammatory foci of the neonatal lung. Mucosal. Immunol..

[44] Farrell H.E., Lawler C., Oliveira M.T., Davis-Poynter N., Stevenson P.G. (2015). Alveolar Macrophages Are a Prominent but Nonessential Target for Murine Cytomegalovirus Infecting the Lungs. J. Virol..

[45] Brody A.R., Craighead J.E. (1974). Pathogenesis of pulmonary cytomegalovirus infection in immunosuppressed mice. J. Infect. Dis..

[46] Wagner F.M., Brizic I., Prager A., Trsan T., Arapovic M., Lemmermann N.A., Podlech J., Reddehase M.J., Lemnitzer F., Bosse J.B. (2013). The viral chemokine MCK-2 of murine cytomegalovirus promotes infection as part of a gH/gL/MCK-2 complex. PLoS Pathog..

[47] Lueder Y., Heller K., Ritter C., Keyser K.A., Wagner K., Liu X., Messerle M., Stahl F.R., Halle S., Forster R. (2018). Control of primary mouse cytomegalovirus infection in lung nodular inflammatory foci by cooperation of interferon-gamma expressing CD4 and CD8 T cells. PLoS Pathog..

[48] Ziegler H., Thale R., Lucin P., Muranyi W., Flohr T., Hengel H., Farrell H., Rawlinson W., Koszinowski U.H. (1997). A mouse cytomegalovirus glycoprotein retains MHC class I complexes in the ERGIC/cis-Golgi compartments. Immunity.

[49] Kavanagh D.G., Koszinowski U.H., Hill A.B. (2001). The murine cytomegalovirus immune evasion protein m4/gp34 forms biochemically distinct complexes with class I MHC at the cell surface and in a pre-Golgi compartment. J. Immunol..

[50] Thiel N., Keyser K.A., Lemmermann N.A., Oduro J.D., Wagner K., Elsner C., Halenius A., Lenac Rovis T., Brinkmann M.M., Jonjic S. (2016). The Mouse Cytomegalovirus Gene m42 Targets Surface Expression of the Protein Tyrosine Phosphatase CD45 in Infected Macrophages. PLoS Pathog..

[51] Yunis J., Farrell H.E., Bruce K., Lawler C., Sidenius S., Wyer O., Davis-Poynter N., Stevenson P.G. (2018). Murine cytomegalovirus degrades MHC class II to colonize the salivary glands. PLoS Pathog..

[52] Farrell H.E., Bruce K., Lawler C., Oliveira M., Cardin R., Davis-Poynter N., Stevenson P.G. (2017). Murine Cytomegalovirus Spreads by Dendritic Cell Recirculation. MBio.

[53] Coles M.C., Veiga-Fernandes H., Foster K.E., Norton T., Pagakis S.N., Seddon B., Kioussis D. (2006). Role of T and NK cells and IL7/IL7r interactions during neonatal maturation of lymph nodes. Proc. Natl. Acad. Sci. USA.

[54] Farrell H.E., Lawler C., Tan C.S., MacDonald K., Bruce K., Mach M., Davis-Poynter N., Stevenson P.G. (2016). Murine Cytomegalovirus Exploits Olfaction To Enter New Hosts. MBio.

[55] Sacher T., Podlech J., Mohr C.A., Jordan S., Ruzsics Z., Reddehase M.J., Koszinowski U.H. (2008). The major virus-producing cell type during murine cytomegalovirus infection, the hepatocyte, is not the source of virus dissemination in the host. Cell Host Microbe.

[56] Teichert M., Milde L., Holm A., Stanicek L., Gengenbacher N., Savant S., Ruckdeschel T., Hasanov Z., Srivastava K., Hu J. (2017). Pericyte-expressed Tie2 controls angiogenesis and vessel maturation. Nat. Commun..

[57] Holtappels R., Podlech J., Geginat G., Steffens H.P., Thomas D., Reddehase M.J. (1998). Control of murine cytomegalovirus in the lungs: Relative but not absolute immunodominance of the immediate-early 1 nonapeptide during the antiviral cytolytic T-lymphocyte response in pulmonary infiltrates. J. Virol..

[58] Krmpotic A., Bubic I., Polic B., Lucin P., Jonjic S. (2003). Pathogenesis of murine cytomegalovirus infection. Microbes Infect..

[59] Travis W.D., Colby T.V., Koss M.N., Rosado-de-Christenson M.L., Müller N.L., King T.E. (2002). Lung Infections. Non-Neoplastic Disorders of the Lower Respiratory Tract (AFIP Atlas of Nontumor Pathology Series Vol. 2).

[60] Khairallah C., Netzer S., Villacreces A., Juzan M., Rousseau B., Dulanto S., Giese A., Costet P., Praloran V., Moreau J.F. (2015). Gammadelta T cells confer protection against murine cytomegalovirus (MCMV). PLoS Pathog..

[61] Seckert C.K., Grieβl M., Büttner J.K., Freitag K., Lemmermann N.A.W., Hummel M.A., Liu X.-F., Abecassis M.I., Angulo A., Messerle M., Reddehase M.J. (2013). Immune Surveillance of Cytomegalovirus Latency and Reactivation in Murine Models: Link to ‘Memory Inflation’. Cytomegaloviruses: From Molecular Pathogenesis to Intervention.

[62] Wong M.T., Ong D.E., Lim F.S., Teng K.W., McGovern N., Narayanan S., Ho W.Q., Cerny D., Tan H.K., Anicete R. (2016). A High-Dimensional Atlas of Human T Cell Diversity Reveals Tissue-Specific Trafficking and Cytokine Signatures. Immunity.

[63] Reddehase M.J., Mutter W., Munch K., Buhring H.J., Koszinowski U.H. (1987). CD8-positive T lymphocytes specific for murine cytomegalovirus immediate-early antigens mediate protective immunity. J. Virol..

[64] Podlech J., Holtappels R., Wirtz N., Steffens H.P., Reddehase M.J. (1998). Reconstitution of CD8 T cells is essential for the prevention of multiple-organ cytomegalovirus histopathology after bone marrow transplantation. J. Gen. Virol..

[65] Holtappels R., Lemmermann N.A., Podlech J., Ebert S., Reddehase M.J. (2016). Reconstitution of CD8 T Cells Protective against Cytomegalovirus in a Mouse Model of Hematopoietic Cell Transplantation: Dynamics and Inessentiality of Epitope Immunodominance. Front. Immunol..

[66] Jonjic S., Mutter W., Weiland F., Reddehase M.J., Koszinowski U.H. (1989). Site-restricted persistent cytomegalovirus infection after selective long-term depletion of CD4^+^ T lymphocytes. J. Exp. Med..

[67] Jonjic S., Pavic I., Lucin P., Rukavina D., Koszinowski U.H. (1990). Efficacious control of cytomegalovirus infection after long-term depletion of CD8^+^ T lymphocytes. J. Virol..

[68] Walton S.M., Wyrsch P., Munks M.W., Zimmermann A., Hengel H., Hill A.B., Oxenius A. (2008). The dynamics of mouse cytomegalovirus-specific CD4 T cell responses during acute and latent infection. J. Immunol..

[69] Mandaric S., Walton S.M., Rulicke T., Richter K., Girard-Madoux M.J., Clausen B.E., Zurunic A., Kamanaka M., Flavell R.A., Jonjic S. (2012). IL-10 suppression of NK/DC crosstalk leads to poor priming of MCMV-specific CD4 T cells and prolonged MCMV persistence. PLoS Pathog..

[70] Jeitziner S.M., Walton S.M., Torti N., Oxenius A. (2013). Adoptive transfer of cytomegalovirus-specific effector CD4^+^ T cells provides antiviral protection from murine CMV infection. Eur. J. Immunol..

[71] Bukowski J.F., Woda B.A., Welsh R.M. (1984). Pathogenesis of murine cytomegalovirus infection in natural killer cell-depleted mice. J. Virol..

[72] Sumaria N., van Dommelen S.L., Andoniou C.E., Smyth M.J., Scalzo A.A., Degli-Esposti M.A. (2009). The roles of interferon-gamma and perforin in antiviral immunity in mice that differ in genetically determined NK-cell-mediated antiviral activity. Immunol. Cell Biol..

[73] Sell S., Dietz M., Schneider A., Holtappels R., Mach M., Winkler T.H. (2015). Control of murine cytomegalovirus infection by gammadelta T cells. PLoS Pathog..

[74] Stacey M.A., Marsden M., Pham N.T., Clare S., Dolton G., Stack G., Jones E., Klenerman P., Gallimore A.M., Taylor P.R. (2014). Neutrophils recruited by IL-22 in peripheral tissues function as TRAIL-dependent antiviral effectors against MCMV. Cell Host Microbe.

[75] Klenovsek K., Weisel F., Schneider A., Appelt U., Jonjic S., Messerle M., Bradel-Tretheway B., Winkler T.H., Mach M. (2007). Protection from CMV infection in immunodeficient hosts by adoptive transfer of memory B cells. Blood.

[76] Ebert S., Becker M., Lemmermann N.A., Buttner J.K., Michel A., Taube C., Podlech J., Bohm V., Freitag K., Thomas D. (2014). Mast cells expedite control of pulmonary murine cytomegalovirus infection by enhancing the recruitment of protective CD8 T cells to the lungs. PLoS Pathog..

[77] Shanley J.D., Pesanti E.L., Nugent K.M. (1982). The pathogenesis of pneumonitis due to murine cytomegalovirus. J. Infect. Dis..

[78] Oduro J.D., Redeker A., Lemmermann N.A., Ebermann L., Marandu T.F., Dekhtiarenko I., Holzki J.K., Busch D.H., Arens R., Cicin-Sain L. (2016). Murine cytomegalovirus (CMV) infection via the intranasal route offers a robust model of immunity upon mucosal CMV infection. J. Gen. Virol..

[79] Zhang S., Caldeira-Dantas S., Smith C.J., Snyder C.M. (2019). Persistent viral replication and the development of T-cell responses after intranasal infection by MCMV. Med. Microbiol. Immunol..

[80] Shanley J.D., Thrall R.S., Forman S.J. (1997). Murine cytomegalovirus replication in the lungs of athymic BALB/c nude mice. J. Infect. Dis..

[81] Reusch U., Muranyi W., Lucin P., Burgert H.G., Hengel H., Koszinowski U.H. (1999). A cytomegalovirus glycoprotein re-routes MHC class I complexes to lysosomes for degradation. EMBO J..

[82] Halle S., Keyser K.A., Stahl F.R., Busche A., Marquardt A., Zheng X., Galla M., Heissmeyer V., Heller K., Boelter J. (2016). In Vivo Killing Capacity of Cytotoxic T Cells Is Limited and Involves Dynamic Interactions and T Cell Cooperativity. Immunity.

[83] Lisnic B., Lisnic V.J., Jonjic S. (2015). NK cell interplay with cytomegaloviruses. Curr. Opin. Virol..

[84] Del Rio M.L., Bernhardt G., Rodriguez-Barbosa J.I., Forster R. (2010). Development and functional specialization of CD103^+^ dendritic cells. Immunol. Rev..

[85] Reuter S., Lemmermann N.A.W., Maxeiner J., Podlech J., Beckert H., Freitag K., Teschner D., Ries F., Taube C., Buhl R. (2019). Coincident airway exposure to low-potency allergen and cytomegalovirus sensitizes for allergic airway disease by viral activation of migratory dendritic cells. PLoS Pathog..

[86] Prendergast A.J., Klenerman P., Goulder P.J. (2012). The impact of differential antiviral immunity in children and adults. Nat. Rev. Immunol..

[87] Fitzgerald N.A., Papadimitriou J.M., Shellam G.R. (1990). Cytomegalovirus-induced pneumonitis and myocarditis in newborn mice. A model for perinatal human cytomegalovirus infection. Arch. Virol..

